# Chromosome-level genome assembly of *Odontothrips loti* Haliday (Thysanoptera: Thripidae)

**DOI:** 10.1038/s41597-024-03289-x

**Published:** 2024-05-04

**Authors:** Luo Yingning, Wei Shuhua, Dai Wenting, Miao Miao, Wang Ying, Zhang Rong, Ban Liping

**Affiliations:** 1 College of Grassland Science and Technology, China Agricultural University, Beijing, 100193 China; 2 Institute of Plant Protection, Ningxia Academy of Agriculture and Forestry Sciences, Yinchuan, 750002 China

**Keywords:** Genome assembly algorithms, Agricultural genetics

## Abstract

As the predominant pest of alfalfa, *Odontothrips loti* Haliday causes great damages over the major alfalfa-growing regions of China. The characteristics of strong mobility and fecundity make them develop rapidly in the field and hard to be controlled. There is a shortage of bioinformation and limited genomic resources available of *O. loti* for us to develop novel pest management strategies. In this study, we constructed a chromosome-level reference genome assembly of *O. loti* with a genome size of 346.59 Mb and scaffold N50 length of 18.52 Mb, anchored onto 16 chromosomes and contained 20128 genes, of which 93.59% were functionally annotated. The results of 99.20% complete insecta_odb10 genes in BUSCO analysis, 91.11% short reads mapped to the ref-genome, and the consistent tendency among the thrips in the distribution of gene length reflects the quality of genome. Our study provided the first report of genome for the genus *Odontothrips*, which offers a genomic resource for further investigations on evolution and molecular biology of *O. loti*, contributing to pest management.

## Background & Summary

**Fig. 1:**
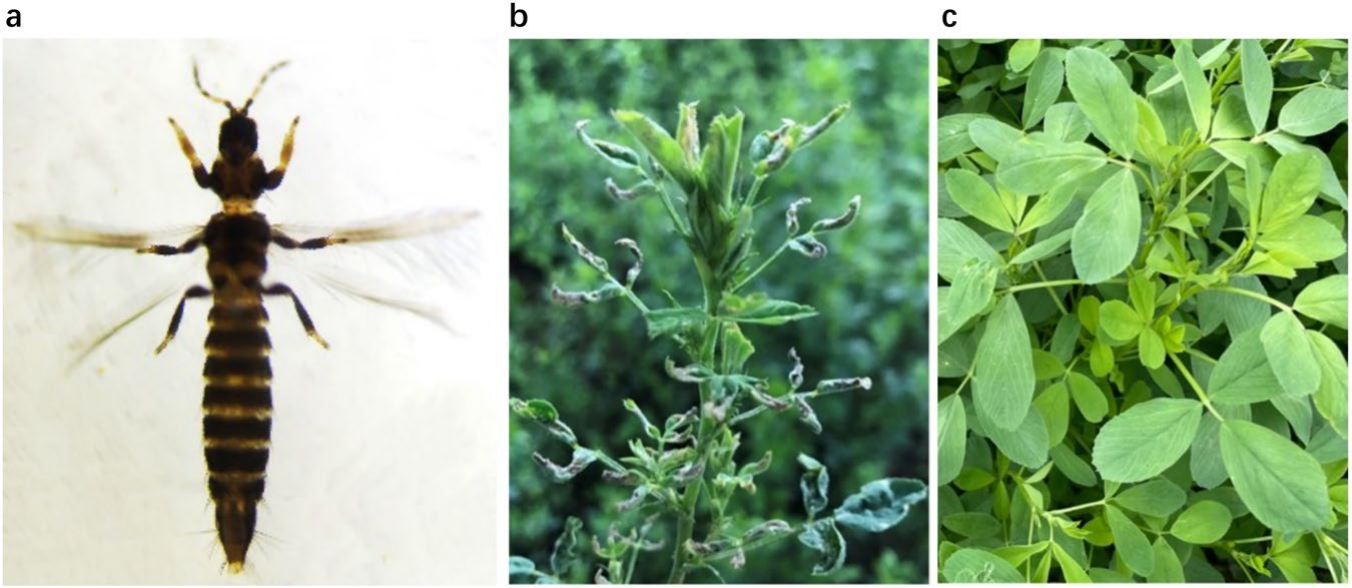
*Odontothrips loti* (**a**), alfalfa with *O. loti* damage (**b**) and without *O. loti* damage (**c**).

*Odontothrips loti* Haliday (Thysanoptera: Thripidae) is a destructive, oligophagous pest that mainly feeds on leguminous crops, particularly alfalfa *Medicago sativa* L.^1,2^. As the predominant pest of alfalfa, in North China, the major alfalfa-growing region, *O. loti* can cause damage to 70%~100% of plants on average^3,4^. Thrips attack the entire life cycle of the host plants, causing the plants to wilt or stop growing and the leaves to turn dry (Fig. 1), which not only leads to severe yield and forage quality reductions but also exacerbates the spread of plant viruses^5–7^. Several features of thrips such as small body size, cryptic behavior, and high fecundity make them difficult to control.

Taking advantages of the low-cost of next generation sequencing (NGS) technology, researchers could identify functional genes related to virus transmission or pesticide resistance from the whole genome level through the construction of genome map, understand the evolution of pesticide resistance and virus transmission mechanisms, and control pest by gene regulation, making it possible to develop new pest management strategies^8–15^. As the genetic information of *O. loti* is still largely unknown currently, we aimed to disclose it for the development of novel *O. loti* control strategies.

In this study, we present a high-quality chromosome-level genome of *O. loti*, which was obtained using a combination of ONT long-read sequencing, Illumina short-read sequencing and chromosome conformation capture (Hi-C) technologies. Comparative genomic analysis was also performed on *O. loti* and another fourteen insect species to explore their phylogenetic relationship and genomic features. We provide the first genome assembly for a thrip in the Odontothrips genus to facilitate better understanding the genome evolution of thrips and developing novel control strategies for this important alfalfa pest.

## Methods

### Sample preparation

*Odontothrips loti* individuals were initially collected from the alfalfa field at Shangzhuang Experimental Station at the China Agricultural University (40°8’15”N, 116°11’18”E), and the colony was established and maintained for approximately 10 generations in the laboratory using the ‘Zhongmu No.1’ alfalfa at the temperature of 25 ± 1 °C, the relative humidity of 65 ± 5%, and the light: dark cycle of 16 h:8 h. The developmental stages of the thrips were examined under a light microscope. Individuals were collected, flash frozen in liquid nitrogen, and stored at −80 °C until use. Detailed information for *O. loti* sampling was shown in Table 1.

**Table 1:** Sample information of *Odontothrips loti* in this study.

Sample	Nymph /Adult	Sex	The number of thrips
DNA for survey	Adult	Female	1
DNA for assembly	Adult	Female and male	800
DNA for Hi-C	Adult	Female and male	800
RNA for annotation	Nymph and adult	Female and male	240

### Genomic DNA sequencing

For Illumina short-read sequencing, the genomic DNA was isolated from of a single female adult following Chen’s protocol^16^, briefly, using sodium dodecyl sulfate (SDS) and proteinase K digestion, followed by phenol-chloroform extraction. The library (150 bp inserts) was constructed with Nextera DNA Flex Library Prep Kit (Illumina, San Diego, CA, USA), and sequenced on the Illumina NovaSeq 6000 (Illumina, San Diego, CA, USA), generating 43.66 Gb of raw data with 150 bp pair-end reads. Adapters and low-quality short reads were removed by Fastp (v0.21.0)^17^ with default parameters, resulting in a total of 42.05 Gb (~123 × coverage) of clean data (Table 2). The short-read data was used for genome survey and assembly polish.

**Table 2:** Library sequencing data and methods used in this study to assemble the *Odontothrips loti* genome.

Sequencing strategy	Platform	Usage	Insertion size	Clean data (Gb)	Coverage (X)
Short-reads	Illumina	Survey Assembly	150 bp	42.05	123
Long-reads	Oxford Nanopore	Assembly	10–20Kb	39.63	116
Hi-C	Illumina	Hi-C assembly	150 bp	31.78	93
RNA-seq	Oxford Nanopore	Annotation	1–15Kb	10.24	30

For long-read genomic DNA sequencing, we used approximately 800 mixed-sex adult thrips. Genomic DNA was extracted using the SDS method^16^, and the DNA fragment size and the degree of degradation were checked on a 0.7% agarose gel. The purity and concentration of extracted DNA were determined with NanoDrop One (Thermo Fisher Scientific). The library was constructed with SQK-LSK109 kit (Oxford Nanopore Technologies, Oxford, UK) according to the manufacturer’s instructions and sequenced on the Oxford Nanopore PromethION platform (Oxford Nanopore Technologies, Oxford, UK). We obtained 41.19 Gb (~120 × coverage) of raw long-read data with mean length of 6,182.26 bp (N50 = 16,150 bp). We then used Oxford Nanopore GUPPY (v0.3.0, https://timkahlke.github.io/LongRead_tutorials/BS_G.html) to filter reads with quality score < 7 and obtained 39.63 Gb (~116 × coverage) of clean reads. The cleaned long-read data were used for contig-level genome assembly (Table 2).

### Hi-C library preparation and sequencing

The Hi-C sequencing library was prepared with 800 mixed-sex adult thrips. Samples were cross-linked with a 2% formaldehyde isolation buffer and then treated with DpnII (New England Biolabs, Beijing, CN) to digest nuclei. Biotinylated nucleotides were used to repair tails, and the ligated DNA was split into fragments of 300–700 bp in length. The resulting Hi-C library was sequenced in Illumina Novoseq 6000 for 150 bp paired-end reads. After applying the same filter criteria for short reads, a total of 31.78 Gb (~93 × coverage) of clean data was generated to assist the chromosome-level assembly (Table 2).

### ONT-Transcriptome sequencing

For ONT-transcriptome sequencing, approximately 240 thrips including nymph and adult were mixed for RNA extraction with the RNA Easy Fast Tissue/Cell Kit (Tiangen). NanoDrop (Thermo Fisher Scientific) and Qubit 3.0 Fluorometer (Life Technologies, Carlsbad, CA, USA) were used to evaluate the quality of extracted RNA. SQK-PCS109 and SQK-PBK004 kit (Oxford Nanopore Technologies) were used for reverse transcript and construction of cDNA library, and sequencing was proceeded on the PromethION sequencer (Oxford Nanopore Technologies, Oxford, UK). A total of 10.24 Gb of clean reads were generated with mean length of 1,034.61 bp (N50 = 1,238 bp), used to assist genome annotation (Table 2).

### Estimation of genomic characteristics

**Fig. 2:**
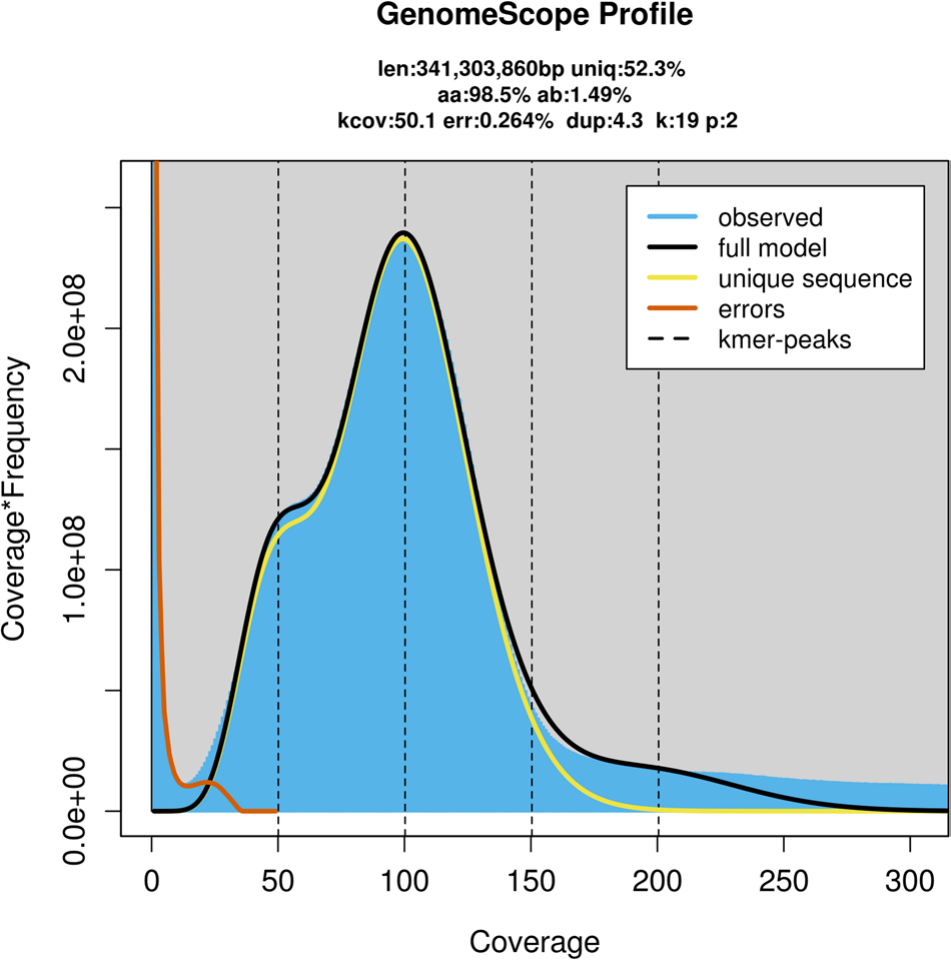
Characteristics of the Illumina short-read sequencing of the *Odontothrips loti* genome.

Genomic characteristics were estimated based on 42.05 Gb of short-read data using a K-mer-based statistical analysis in Jellyfish (v2.3.0)^18^ and GenomeScope2^19^ (p = 2, k = 19). Based on 19-mer depth analysis, the genome size and heterozygosity were estimated to be 341.3 Mb and 1.49%, respectively, therefore, this genome is considered highly heterozygous (Fig. 2).

### Genome assembly

#### Contig level assembly

We first used NextDenovo (v2.5.0)^20^ to generate a draft assembly, and conducted two rounds of polish with ONT long reads on Racon (v1.4.11, https://github.com/lbcb-sci/racon). Illumina reads were mapped to the assembly using BWA v0.7.17 and another two rounds of contig polishing were performed with Pilon (v1.23)^21^. Owing to its highly heterozygous feature, Purge_haplotigs (v1.0.4, https://github.com/skingan/purge_haplotigs_multiBAM) was applied to de-heterozygosis the draft genome to generate the final contig-level genome, which was 346.58-Mb long and similar to the estimated size, with the N50 contig length of 8.59 Mb (Table 3).

**Table 3:** Major indicators of the *Odontothrips loti* genome.

Features	Values
Estimated genome size (bp)	341,303,860
Contig-level assembly size (bp)	346,577,358
Chromosome-level assembly size (bp)	346,592,158
Anchored to chromosome (bp)	301,277,358
Contig N50(bp)	8,588,564
Scaffold N50(bp)	18,519,078

#### Hi-C scaffolding

**Fig. 3:**
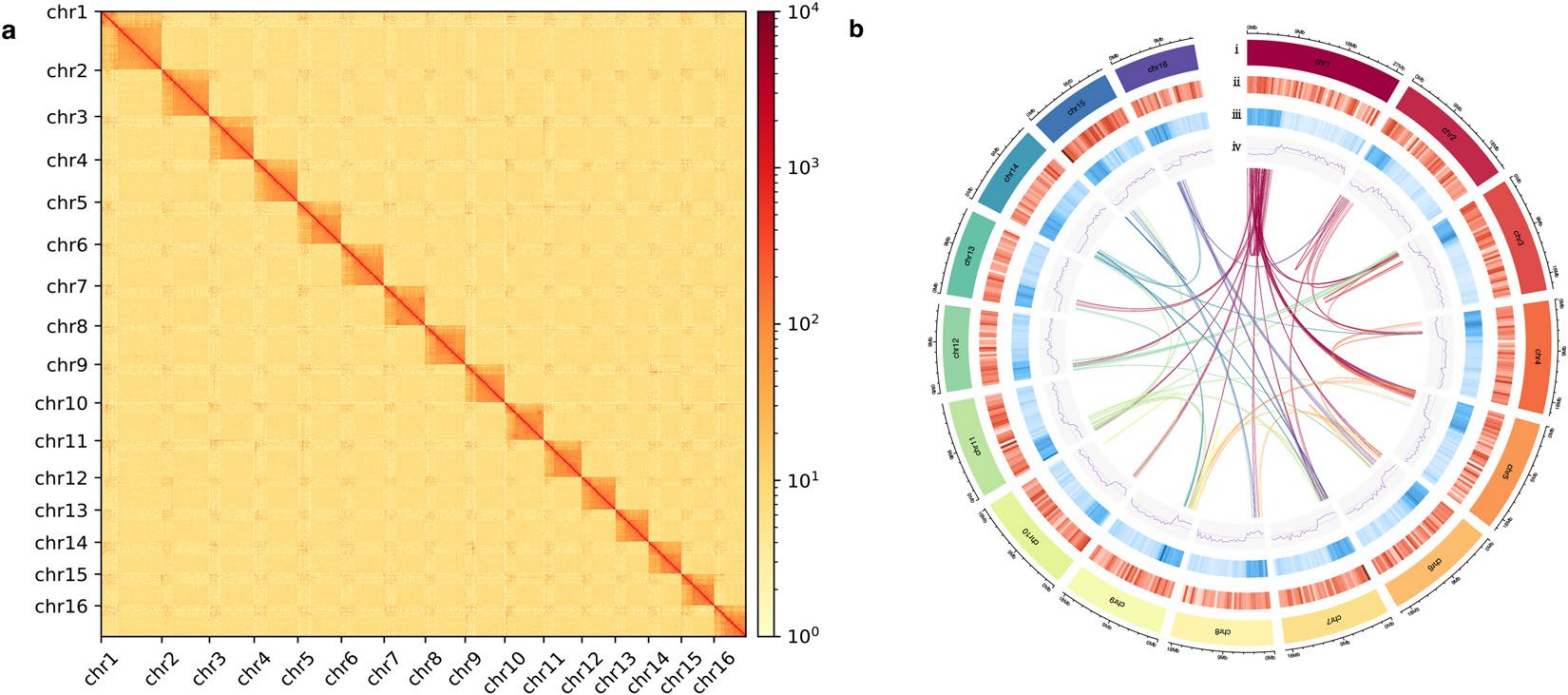
Heatmap of genome-wide Hi-C data and circular representation of the chromosomes of *Odontothrips loti*. (**a**) The heatmap of chromosome interactions in *O. loti*. The frequency of Hi-C interaction links is represented by colors, which ranges from yellow (low) to red (high). (**b**) Circos plot of distribution of the genomic elements in *O. loti*. The tracks indicate (i) length of the chromosome, (ii) gene density, (iii) distribution of transposable element (TE) density, and (iv) GC density. Center: intra-genomic syntenic blocks of *O. loti*. The densities of genes, TEs, and GC were calculated in 500 kb windows.

Low-quality raw reads (quality score <20,length shorter than 30 bp) and adaptors were removed using Fastp (v0.21.0)^17^. The clean reads were then mapped to the contig assembly using HICUP (v0.8.0)^22^ to filter unmapped reads, invalid pairs, dangling end and repeats resulting from PCR amplification. The valid paired-end pairs were used for contig cluster, order and orient by ALLHIC (v0.9.8)^23^. The interaction between contig pairs were converted into binary files by 3D-DNA^24^ and Juicer (v1.6)^25^. The HiCExplorer (v3.6)^26^ was used to generate the heat maps of contig interaction intensity and location. The Juicebox (v1.11.08)^27^ was subsequently employed to review assembly manually. In summary, the resulting chromosome-level genome length was 346.59 Mb with a scaffold N50 of 18.52 Mb (Table 3), around 86.93% (301.28 Mb) of the genome bases were anchored onto 16 chromosomes (Fig. 3a), and most syntenic blocks of genome presents in the low GC content region (Fig. 3b).

### Predicting repeats

We used ReaptModeler (v.1.0.11, https://github.com/Dfam-consortium/RepeatModeler) to predict repeat sequence. LTR_FINDER (vOfficial, -size 1000000 -time 300)^28^ and LTR_retriever (v2.9.0)^29^ were used to find and de-redundant the LTR sequence. These two de novo library were combined with RepBase^30^ for further prediction by RepeatMasker (v4.0.9,-nolow -no_is -norna)^31^. RepeatProteinMask (-noLowSimple -pvalue 0.0001) was used for homo-prediction. All results were de-redundant and merged to the final repeat sequence. In summary, 115.26 Mb repeat sequences were identified, accounting for 33.26% of the *O. loti* genome (Table 4). Among these repeat sequences, most (18.85%) are DNA transposon, followed by 10.13% of long terminal repeats (LTRs), 3.45% of long interspersed nuclear elements (LINEs) and only 0.40% of short interspersed nuclear elements (SINEs) (Table 4).

**Table 4:** Statistics of the repeat sequences annotation in *Odontothrips loti* genome.

Type	Length (bp)	Percentage in genome (%)
DNA	65,317,630	18.85
LTR	35,092,753	10.13
LINE	11,957,062	3.45
SINE	1,382,412	0.40
Unknown	14,723,706	4.25
Total	115,261,572	33.26

### Protein-coding genes and functional predictions

We utilized a pipeline include three strategies: transcriptome-based prediction, homology-based prediction, and ab initio prediction to annotate protein coding genes. For transcriptome-based prediction, we use NanoFilt (v2.8.0, -q 7 -l 100 -headcrop 30 -minGC 0.3)^32^, Pychopper (v2.7.2, https://github.com/epi2me-labs/pychopper), racon (v1.4.11, https://github.com/lbcb-sci/racon), minimap2 (v2.17-r941)^33^, stringtie (v2.1.4)^34^ and TransDecoder (v5.1.0, https://github.com/TransDecoder/TransDecoder) for ONT-transcriptome reads to predicted protein-coding gene. For homology-based prediction, tblastn (v2.7.1)^35^ with an E-value cutoff of 1e-5 and Exonerate (v2.4)^36^ were used to predict gene structure by comparing with 3 closely related species (*Megalurothrips usitatus*, *Thrips palmi*, *Frankliniella occidentalis*) and model species *Drosophila melanogaster*. Before ab initio prediction, repetitive elements from the whole genome were soft-masked. Augustus (v3.3.2)^37^, GenScan (v1.0)^38^ and GlimmerHMM (v3.0.4)^39^ were used for de novo prediction. Finally, MAKER (v2.31.10)^40^ integrated the above three strategies, resulting in a non-redundant gene set, with weighting as default. Overall, 20,128 protein coding genes were obtained (Table 5).

**Table 5:** Statistics for the *Odontothrips loti* functionally annotated protein-coding genes.

Database	Number	Percentage (%)
Protein-coding genes	20,128	100.00
Annotated genes	18,837	93.59
Interproscan	17,895	88.91
NR	16,363	81.29
Uniprot	16,241	80.69
Pfam	13,932	69.22
GO	12,229	60.76
KEGG	8,527	42.36
Pathway	4,801	23.85
Unanotated genes	1,291	6.41

For functional annotation, protein sequences were aligned to Non-Redundant protein (NR), Universal Protein (Uniprot), Protein Families Analysis and Modeling (Pfam), Clusters of Orthologous Groups of proteins (COG), Kyoto Encyclopedia of Genes and Genomes (KEGG) and evolutionary genealogy of genes: Non-supervised Orthologous Groups (eggNOG) database. Gene Ontology (GO) terms was obtained from Uniport. InterProScan (v5.52-86.0)^41^ was used to search the conserved sequences, motifs and domains. There were 12,229 (60.76%) and 8,527 (42.36%) genes annotated to GO terms and KEGG pathways respectively. A total of 18,837 genes (93.59%) were annotated using at least one public database (Table 5).

## Data Records

The assembly genome sequence and annotation data were deposited in Figshare^42^ and GenBank^43^. Raw data from Nanopore (CRR997575)^44^, Illumina (CRR997573)^45^ and Hi-C (CRR997574)^46^ genome sequencing and RNA-seq (CRR997576)^47^ were deposited in the Genome Sequence Archive (GSA, https://ngdc.cncb.ac.cn/gsa)^48^, and were related to the BioProject PRJCA022165.

## Technical Validation

### Genome quality assessment

**Fig. 4:**
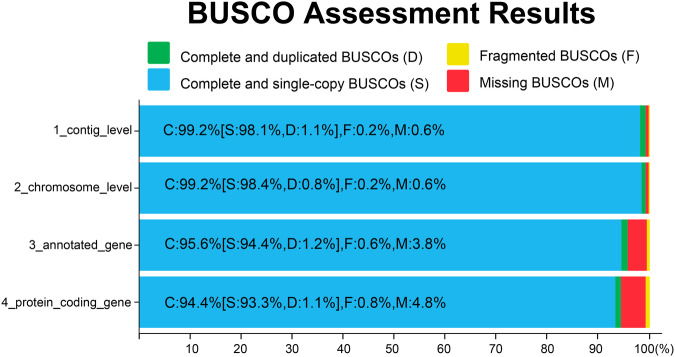
Benchmarking of genome completeness of *Odontothrips loti* genome assembly and annotation, evaluated by BUSCO based on insect_odb10 database which includes 1,367 genes. C: the number of complete genes, S: the number of complete and single-copy genes, D: the number of complete and duplicated genes, F: the number of incomplete genes, M: the number of missing genes.

We assessed the quality of chromosome-level genome from the three aspects: continuity, consistency, and completeness. First, the scaffold N50 of *O. loti* is 18.52 Mb (Table 3), representing the continuity of genome. Second, we evaluated the consistency of the genome by calculating the comparison rate and coverage of Illumina reads through BWA (v0.7.17)^49^, resulting 91.11% short reads were aligned to and covered 94.68% of the ref-genome. Third, we used BUSCO (v4.1.4)^50^ to estimate the completeness of chromosome-level genome by searching the 1367 BUSCO genes in insecta_odb10 (https://busco-data.ezlab.org/v5/data/lineages/). The results showed a high completeness level with 99.2%, 99.2%, 95.6%, 94.4% complete genes found in the contig-level genome, chromosome-level genome, annotated gene sets and protein-coding gene sets, respectively (Fig. 4).

### Evaluation of gene prediction

**Fig. 5:**
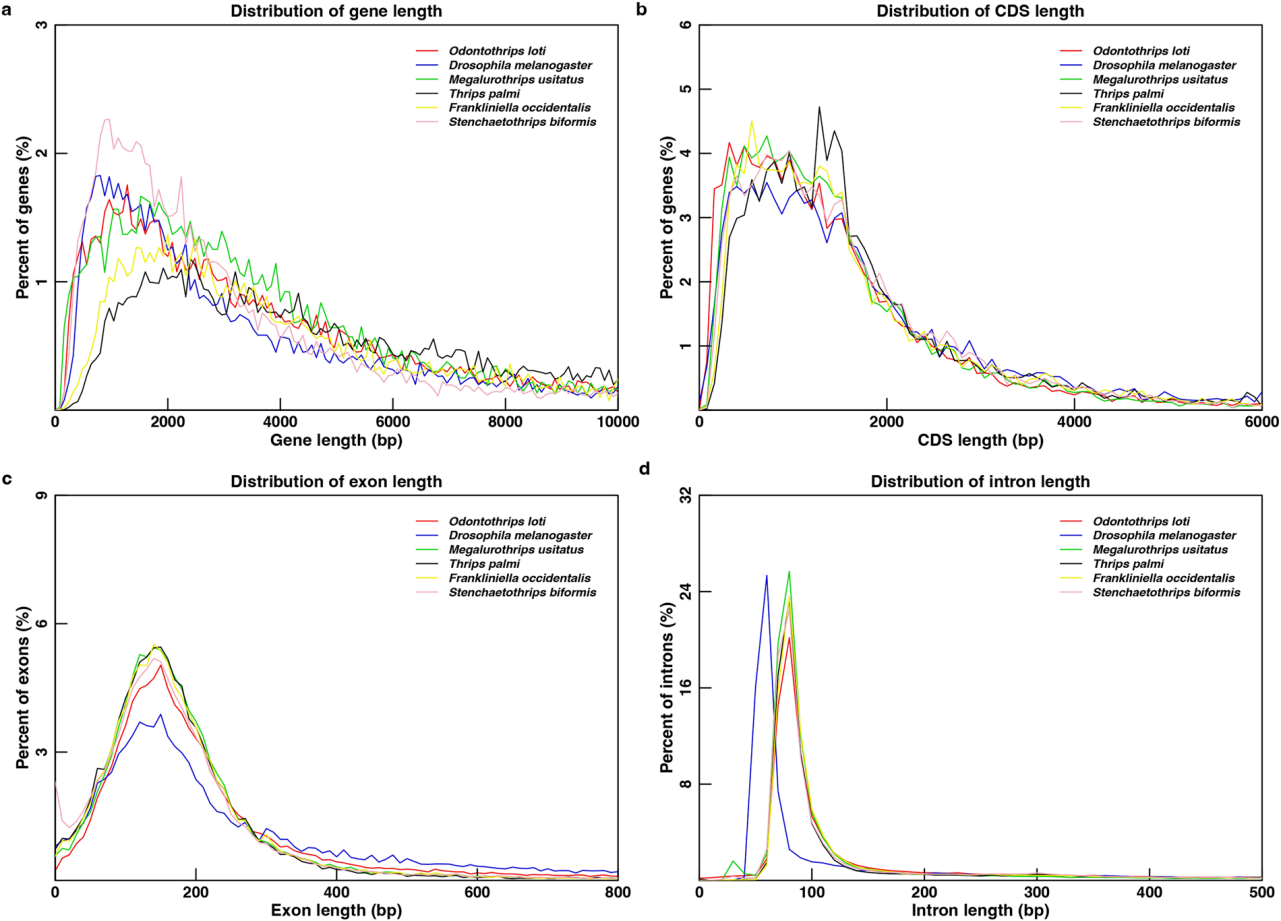
Annotated genes comparison of the distribution of (**a**) gene length (**b**) CDS length (**c**) exon length (**d**) intron length in *Odontothrips loti* with *Drosophila melanogaster* and four closely related species. The x-axis represents the length, and the y-axis represents the density of genes.

To verify the accuracy and reliability of the gene prediction, we determined the distribution of gene length, CDS length, exon length and intron length in *O. loti*, *D. melanogaster*^51^ and other four related species (*M. usitatus*^8^, *T. palmi*^12^, *F. occidentalis*^14^*, S. biformis*^13^). The consistent tendency among the thrips supported an ideal annotated gene dataset in *O. loti* (Fig. 5).

## Data Availability

All software and pipelines were executed according to the manual and protocols of the published bioinformatic tools. The version and code/parameters of software have been described in Methods section. No custom code was used.
